# BCG vaccination decline and pediatric tuberculosis rise in Brazil: spatial-temporal study

**DOI:** 10.11606/s1518-8787.2025059006458

**Published:** 2025-04-28

**Authors:** Fabiana Rabe Carvalho, Alice Ramos-Silva, Debora Castanheira Pires, Carla Veras Yigashira Oliveira, Claudete Aparecida Araújo Cardoso, Clemax Couto Sant’Anna, Elisangela Costa Lima, Andrea Alice Silva

**Affiliations:** I Universidade Federal Fluminense. Faculdade de Medicina. Laboratório Multiusuário de Apoio à Pesquisa em Nefrologia e Ciências Médicas. Niterói, RJ, Brasil; II Universidade Federal do Rio de Janeiro. Faculdade de Farmácia. Rio de Janeiro, RJ, Brasil; III Instituto Nacional de Infectologia Evandro Chagas. Laboratório de pesquisas clínicas em IST e Aids. Rio de Janeiro, RJ, Brasil; IV Universidade Federal Fluminense. Faculdade de Medicina. Programa de Pós-graduação em Ciências Médicas. Niterói, RJ, Brasil; V Universidade Federal do Rio de Janeiro. Programa de Pós-graduação em Clínica Médica. Rio de Janeiro, RJ, Brasil; VI Universidade Federal Fluminense. Faculdade de Medicina. Programa de Pós-graduação em Patologia. Niterói, RJ, Brasil

**Keywords:** Tuberculosis, Pediatrics, Spatial Analysis, BCG Vaccine, Brazil

## Abstract

To explore the spatial distribution of bacille Calmette-Guerin (BCG) vaccination coverage and tuberculosis cases in Brazilian children under the age of ten in the last two decades.

This is a population-based ecological study using data from the Sistema de Informação de Agravos de Notificação (SINAN – Information System for Notifiable Diseases) from January 2001 to December 2022. We investigated tuberculosis incidence and vaccination coverage in all Brazilian municipalities. Spatial dependence was analyzed by the Global Moran Index and Local Indicators of Spatial Association (LISA). Joinpoint regression was used to assess trends in tuberculosis and BCG rates over time.

The results indicated 39,840 pediatric tuberculosis cases, with 60.65% in children under the age of five. The highest incidence rates were in the states of Amazonas, Mato Grosso, and Rio de Janeiro. Spatial analysis showed significant positive spatial autocorrelation in pediatric tuberculosis cases, with high-high clusters in several states and municipalities. Tuberculosis cases in children under five have been declining since 2000 but rose in 2020 (APC = 26.64; p-value = 0.032). An earlier increase was observed in children up to ten years old, starting six years prior.

The study highlights the decline in BCG vaccination coverage below 90%, with the most significant drop in 2020, particularly in the Northeast. The findings underscore the need for high maintain vaccination coverage and robust public health policies to mitigate tuberculosis in children. Addressing these issues requires targeted public health interventions, especially in regions with higher tuberculosis burdens.

## INTRODUCTION

Tuberculosis disproportionately affects impoverished communities, highlighting the complex interplay between socioeconomic status and disease prevalence^1^. Despite being a BRICS country, Brazil exhibits pronounced social inequalities, and it is designated by the World Health Organization (WHO) as one of the 30 high-burden tuberculosis countries, thus facing health challenges associated with this infectious disease, particularly in pediatric populations^2 , 3^. Although previous research has identified geographic clusters of tuberculosis cases across Brazil, the focus on pediatric tuberculosis has been limited^4 , 5^. Some of the challenges in estimating the burden of pediatric tuberculosis are the absence of a standardized case definition, the difficulty of establishing definitive diagnoses in young children, and the generally lower public health prioritization of pediatric tuberculosis compared to adult cases^6 , 7^.

Children are primarily exposed to tuberculosis through close contact with infected adults within households^6 , 7^. The closure of daycare centers and schools during the COVID-19 pandemic, as well as the discouragement of outside activities by the Brazilian Ministry of Health^7^, may have inadvertently increased the risk of tuberculosis transmission among children, reflecting a possible outbreak in pediatric tuberculosis cases during periods of social isolation^8^.

On the other hand, Brazil’s national immunization program, which provides free vaccines via the Brazilian Unified Health system (SUS), plays a crucial role in managing infectious diseases in children. The bacillus Calmette-Guérin (BCG) vaccine (which is mandatory for Brazilian children under one year since the 1980s) is effective in preventing severe forms of tuberculosis (miliary and meningitis) among this demographic but does not confer similar protection to adolescents and adults^9^. Vaccination rates have declined in recent year, particularly among infants, and research suggests regional disparities in vaccine coverage^10^.

In 2015, the WHO launched the End TB initiative, setting a goal to reduce the tuberculosis incidence rate by 90% by 2035, using the 2015 rate as a baseline^7^.The success of the End TB initiative depends on national commitments and the implementation of effective public health policies^11^. This study aims to explore the spatial distribution of BCG vaccination coverage and tuberculosis cases in Brazilian children up to ten years of age over the past 21 years, seeking to understand the effectiveness of current strategies and identify areas for policy enhancement.

## METHODS

### Study Design

We conducted a population-based ecological study to analyze data from Brazilian children registered in the *Sistema de Informação de Agravos de Notificação* (SINAN – Information System for Notifiable Diseases) from January 2001 to December 2022. Recent systematic reviews indicate that BCG vaccination only significantly reduces severe forms of tuberculosis incidence in children under the age of five^9^. Thus, we focused our subgroup analysis on this age demographic. The spatial scope of our analysis encompassed all municipalities in Brazil, allowing us to comprehensively examine the relationship degree between tuberculosis incidence and vaccination coverage. Our analysis used data on the residences of these children to capture social vulnerability as it relates to geographic location.

### Study Area

Brazil has a territory of 8.5 million square kilometers and approximately 203 million inhabitants. Around 13% (26,454,300) are children under the age of ten, and 6.3% (12,704,860) are under five. The country is organized into five macro-regions, divided into 27 federative units, and subdivided into 5,570 municipalities.

### Participants

Tuberculosis reported to SINAN from 2001 to 2022 in children under ten years of age in 5,570 Brazilian municipalities. No exclusion criteria are applied.

### Variables and Data Sources

In this study, we used the following variables to analyze BCG vaccination coverage and the incidence of tuberculosis among Brazilian children: (i) Tuberculosis cases: the number of new confirmed cases of tuberculosis per 100 thousand children, in the population residing in a given geographic space, was extracted from SINAN. Cases were classified as either pulmonary or extrapulmonary; (ii) Vaccination coverage: this is calculated as the number of doses of the BCG vaccine administered divided by the number of live births in the corresponding year and location. Coverage exceeding 120% was flagged as inconsistent and was analyzed separately to investigate potential data entry errors or reporting issues. Vaccination coverage rates exceeding 100% for BCG vaccine may occur due to inconsistencies in data recording, inaccurate population estimates, and vaccination of children outside their area of residence.

Data on BCG doses, the number of live births, and tuberculosis cases were extracted from Datasus (https://datasus.saude.gov.br/); (iii) Age stratification: children were stratified into two age groups: under five years old and under ten years old. This categorization was based on a recent systematic review, which indicated that BCG vaccination is effective only in children under the age of five^9^; (iv) Spatial data: includes the geographic locations of reported tuberculosis cases and vaccination data, using the Instituto Brasileiro de Geografia e Estatística (IBGE) cartographic base of Brazil; (v) Population data: obtained from the IBGE (https://www.ibge.gov.br/), stratified by age, and used to calculate accurate tuberculosis incidence rates and vaccination coverage per 100,000 children; (vi) Ethnicity data: reported by the patient or their legal guardian and recorded during notification to SINAN.

### Data Analysis

Variables were expressed in absolute and relative frequencies. The spatial distribution maps were analyzed using R software (version 4.3.2), using the cartographic base of Brazil available on the IBGE website. We investigated spatial dependence, which is the tendency of natural or human events to influence each other in terms of proximity, as well as spatial autocorrelation, which quantifies this influence using specific metrics.

We applied the first-order neighborhood matrix “w,” defining as neighbors municipalities with direct borders, and assumed that directly connected regions interact more than those that are not connected. This was represented by a matrix where the value 1 indicates shared borders and 0 indicates no direct connection.

The initial investigation of spatial autocorrelation was conducted through the Global Moran’s Index (I). The spatial cluster’s composition was obtained through the Local Indicators of Spatial Association (LISA). This index enabled the detection of clusters

of areas with similar attributes, which were visualized on the Moran map. This map presents “high-high” areas marked in red indicating municipalities with high cases and positive influence on their neighbors, while “low-low” in green signaled municipalities with low vaccination coverage affecting their neighbors^12^. Areas without significant spatial autocorrelation were presented in gray, indicating statistical significance (p ≤ 0.05). The study adopted a significance level of 5%.

This study employed joinpoint regression to assess trends in tuberculosis and BCG rates over time. This technique models the series using a point-by-point regression method, where the variation is estimated using Poisson regression and the significance tests for the change in trend use the Monte Carlo permutation method. The data were analyzed under the assumption of constant variance (homoscedasticity) and first-order autocorrelation. An annual percent change (APC) in the tuberculosis trend for each line segment was also estimated. The rates are assumed to change at a constant percentage annually. The APC was tested to determine whether there was a difference from this null hypothesis. In the final model, each Joinpoint informs a statistically significant change in trends (increase or decrease), and each of those trends is described by an APC^13^. Therefore, in the final model, each junction point reports a statistically significant change in the trends (increase or decrease), and each of these trends is described by a coefficient (slope)^13^. The analyses were conducted using Joinpoint Regression Program version 4.9.0.0, a trend analysis software developed by the US National Cancer Institute (US NCI) to analyze data from the Surveillance Epidemiology and End Results Program (SEER).

In accordance with Brazilian law, the use of secondary health data, where the identification of individuals or institutions is not possible, does not require approval from the ethics committee.

## RESULTS

In the last two decades, the Brazilian Ministry of Health documented 39,840 cases of pediatric tuberculosis in children under ten years old, 60.65% of which were in children under five. In terms of ethnicity/skin color, Brown/Mixed-race children constituted the largest demographic group, with 15,342 cases (38.5%), followed by 10,076 White children (25.3%); 3,475 Black children (8.7%); 2,112 (5.3%) Indigenous and 255 (0.6%) Asian children; 8,580 children (21.6%) did not have information in the database regarding their ethnicity. A total of 28,159 tuberculosis reports (70.7%) were clinically characterized by pulmonary manifestations.

**Figure 1.**
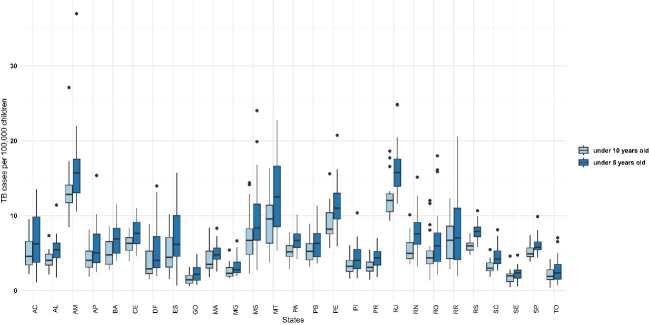
State-wise distribution of tuberculosis incidence among children under ten and five years of age in Brazil, stratified by age group (2001–2022).

AC: Acre; AL: Alagoas; AM: Amazonas; AP: Amapá; BA: Bahia; CE: Ceará; DF: Distrito Federal; ES: Espírito Santo; GO: Goiás; MA: Maranhão; MG: Minas Gerais; MS: Mato Grosso do Sul; MT: Mato Grosso; PA: Pará; PB: Paraíba; PE: Pernambuco; PI: Piauí; PR: Paraná; RJ: Rio de Janeiro; RN: Rio Grande do Norte; RO: Rondônia; RR: Roraima; RS: Rio Grande do Sul; SC: Santa Catarina; SE: Sergipe; SP: São Paulo; TO: Tocantins.

During the study period, the incidence of new tuberculosis cases per 100,000 children under ten varied across Brazilian regions, showing significant heterogeneity among states. The incidence rates were 8.86 in the North, 7.45 in the Southeast, 7.18 in the Northeast, 6.54 in the Midwest and 5.76 in the South. Among the total 5,570 municipalities in Brazil, 2,693 reported at least one new case of tuberculosis in children under the age of ten each year, representing 48.3% of the total Brazilian territory. Furthermore, when evaluating the group at the highest risk of developing tuberculosis and meningitis due to age-related immaturity or compromised nutritional status (< 5 years), we observed a high incidence of tuberculosis cases in all states evaluated, mainly in Amazonas, Mato Grosso, and Rio de Janeiro. Figure 1 illustrates tuberculosis cases per 100,000 children under ten and five years old in Brazilian states.

**Figure 2.**
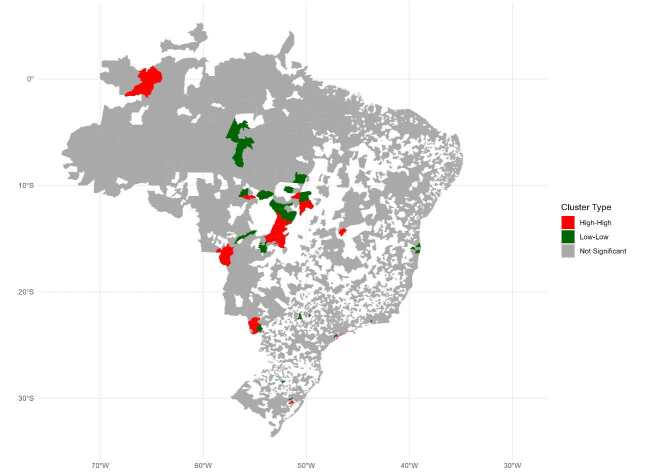
Geographic clustering of pediatric tuberculosis cases in Brazil (under ten years of age).

Note: Red represents areas with a high incidence of geographically correlated cases, green indicates areas with a low incidence of geographically correlated cases, and gray denotes areas with no spatial correlation.

The Global Moran’s index analysis revealed a significantly positive spatial autocorrelation in the distribution of pediatric tuberculosis cases in Brazilian municipalities from 2001 to 2022 (Moran’s I: 0.09; p = 0.001). From 2001 to 2022, 30 clusters with high incidences of pediatric tuberculosis in children under ten years of age were identified in several regions of Brazil. Mato Grosso had the largest number of clusters (n = 11), followed by Mato Grosso do Sul with seven, Goiás with four, and São Paulo with three. Other states worth mentioning are Rio Grande do Sul with two clusters, and Amazonas, Tocantins, and Pernambuco with one cluster each (Figure 2).

**Figure 3.**
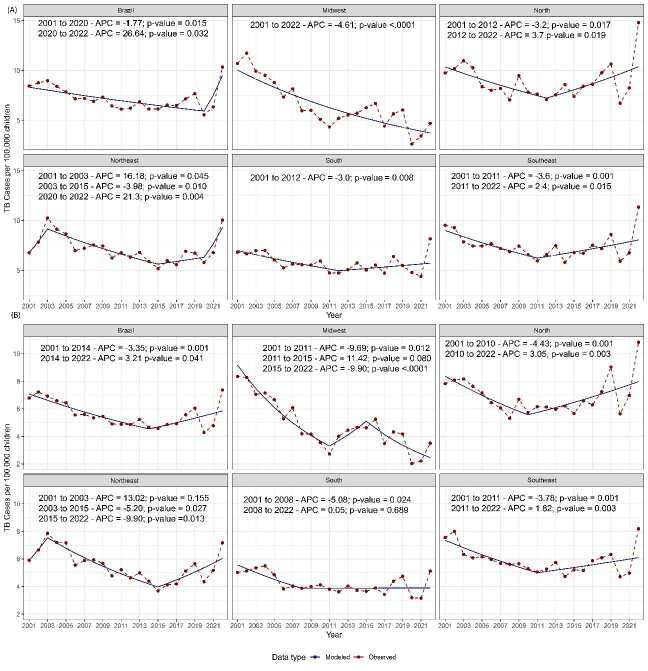
Joinpoint regression indicating the tuberculosis rate in children from 2000 to 2020 per Brazilian region (A) data about children under five; (B) data about children under ten.

The reports suggested a decrease in the number of tuberculosis cases in children under five since 2000.This decline was markedly interrupted in 2020, after which an increasing trend in the incidence of cases within this age group was observed (APC = 26.64; p-value = 0.032). Additionally, when considering children up to ten years of age, an increase in the number of cases was noted six years earlier than that observed in children under five years of age (APC = 3.21; p-value = 0.041) (Figure 3).

**Figure 4.**
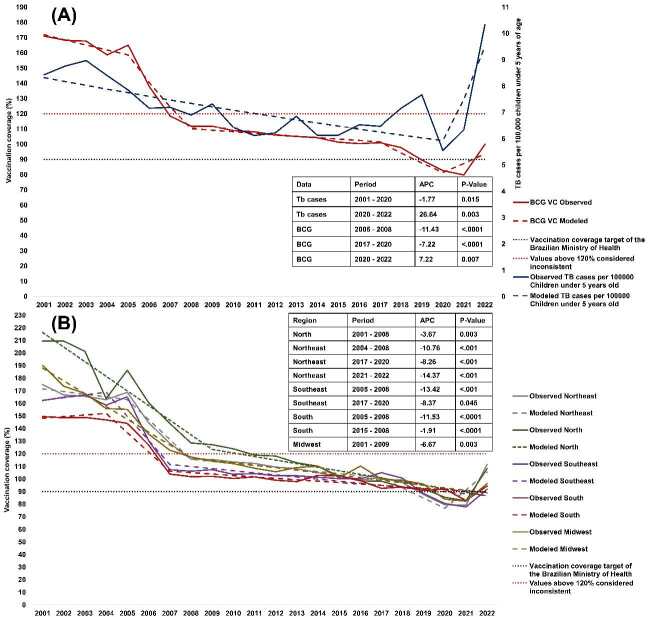
Historical Joinpoint regression analysis indicating vaccination coverage for bacille Calmette-Guerin (BCG VC) and tuberculosis cases for children under five years of age from 2001 to 2022 in Brazil: (A) National data on vaccination coverage and tuberculosis cases in children under five years of age. Right axis –tuberculosis cases per 100,000 children under five years of age; Left axis - BCG vaccination coverage (B) Vaccination coverage for BCG by region.

Figure 4 presents the BCG vaccination coverage trends alongside tuberculosis rates in children under five years of age. In 2020, vaccination coverage dropped below 90% in nearly all Brazilian regions, coinciding with an increase in tuberculosis cases from 2020 onward.

APC: annual percentage change.

**Figure 5.**
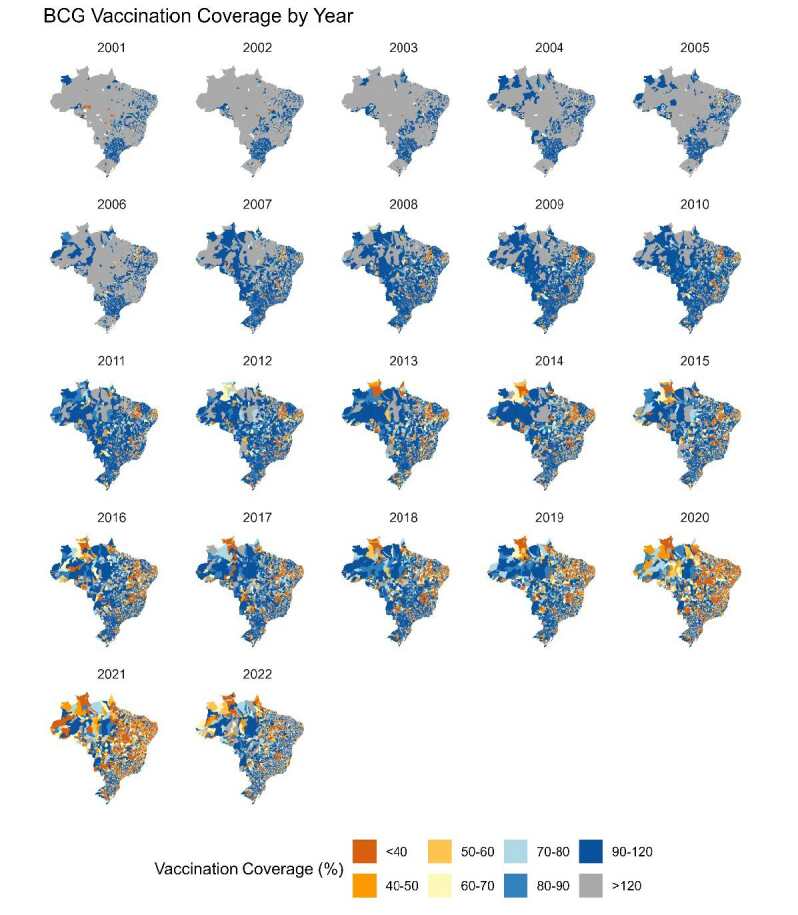
Annual spatial analysis of BCG vaccination coverage in children from 2001 to 2022, by state.

The number of slopes is determined by the number of junction points identified by the analysis. A = BCG vaccination coverage (VC) by region; B = BCG vaccination coverage in Brazil *versus* tuberculosis cases in children under five years old. Right y-axis - tuberculosis cases; left y-axis BCG VC (Figure 5).

BCG: bacille Calmette-Guerin.

Note: The figure illustrates BCG vaccination coverage across Brazil from 2001 to 2022. Vaccination coverage is categorized as follows: less than 40% in brown, 40–50% in orange, 50–60% in light yellow, 60–70% in yellow, 70–80% in light blue, 80–90% in blue, 90–120% in dark blue, and over 120% in gray.

## DISCUSSION

This study assessed segmented trends in the incidence and spatial distribution of new tuberculosis cases in children under ten years of age, as well as the BCG vaccination coverage over the past 21 years. This period was chosen to illustrate the landscape of BCG vaccination and the epidemiological profile of pediatric tuberculosis over the past two decades in Brazil. Compared to other countries with high tuberculosis burden, Brazil has similar rates to Kenya with an average of 75,000 notifications per year. India, Philippines, and Indonesia lead the ranking with more than 300,000 notifications yearly^3^. The literature suggests that social determinants of health play a significant role in the burden of tuberculosis^1^. Our results show spatial autocorrelation in tuberculosis cases among children.

The regional analysis presented a greater concentration of cases in the Amazonas, Rio de Janeiro, and Mato Grosso states. Amazonas concentrates 18% of Brazil’s Indigenous population, according to the IBGE. This population is four times more vulnerable to tuberculosis than the general population, probably due to unfavorable socioeconomic conditions and previously described immunological susceptibility^1 , 14^. Health posts located far from villages requires specific travel logistics, which may increase transmissibility and hinder the monitoring of Indigenous children^14^. Understanding the distribution of tuberculosis cases among children is crucial for guiding treatment and prevention efforts, as well as for developing strategies to address disparities in tuberculosis incidence and mortality^15^.

High-incidence clusters are observed in municipalities near São Gabriel da Cachoeira, in the state of Amazonas, an area close to the borders with Colombia and Venezuela^16^. The issue of immigrants and refugees must be approached ethically, as these individuals, often displaced by adverse conditions, seek refuge in Brazil^17^. According to the principle of universality of the SUS, it is essential that all individuals, including immigrants and refugees, have full access to healthcare. The influx of people into Brazil may influence the region’s epidemiological profile, particularly in border areas with Colombia and Venezuela^18^.

The socioeconomic vulnerability of immigrants and refugees needs to be addressed with a robust and compassionate public health response. Displaced populations face significant barriers to accessing health services, which can exacerbate the spread of infectious diseases, including tuberculosis^19 , 20^. As such, ensuring adequate access to health services, including tuberculosis prevention and treatment, is not only an ethical responsibility but also a critical health need. Implementing programs that consider the specific needs of refugees can improve health outcomes and strengthen the global health response. Moreover, an inclusive approach aids in controlling the spread of tuberculosis, benefiting both refugees and the local population^3^.

The underreporting of pediatric and adolescent tuberculosis is also an important challenge in low- and middle-income countries, exacerbated by the covid-19 pandemic^21^. The observed increase in tuberculosis cases in Brazil since 2020 highlights the need for enhanced epidemiological surveillance and robust public health policies to address more severe cases in this vulnerable population^21^. The overload of health services and the reduction in diagnostic activities due to social distancing measures have likely contributed to the underreporting of tuberculosis cases, potentially affecting vaccination rates^22^.

BCG vaccination is a strategy for reducing severe tuberculosis cases in children from high-burden countries^3^. As Brazil is one of the 30 countries most burdened by tuberculosis, maintaining vaccination coverage above 90% is essential for reducing cases and complications of childhood tuberculosis, such as meningitis and miliary tuberculosis^3^. Despite this, historical data from the past 21 years reveal a persistent decline in BCG vaccination coverage below 90% in all regions of Brazil. The lowest coverage rates were observed in 2020, with only the Southern region maintaining a BCG coverage rate above 90%. The Northeast experienced the most expressive decline. During the covid-19 pandemic, several Brazilian health services reported shortages of medication^23^. The Northeast was the region most affected by the drop in vaccination coverage, likely due to the shortage of essential vaccines such as BCG. Furthermore, Arroyo and colleagues identified clusters with low coverage across several vaccines in the North and Northeast of Brazil, revealing a significant heterogeneity in vaccination coverage, including BCG. Their findings raise concerns about the resurgence of diseases preventable by vaccination^24^.

Brazil reported trends in line with the global drop in BCG vaccination coverage from 2017 to 2022, which was below 90%. Nonetheless, the decrease in coverage rates was more pronounced in low- and middle-income countries^25^, which also have higher incidences of tuberculosis in children. The Americas have the second lowest vaccination coverage among continents, with only Africa having a lower rate^25^. Mexico had a BCG vaccination coverage rate lower than 90%, from 2017 to 2020, with 2020 recording the largest historical decline in coverage^26^.

Furthermore, the decline in tuberculosis among children under five continued until 2020, followed by an unexpected increase over the next two years. This temporal pattern was also observed in other studies investigating tuberculosis across different age groups^27 , 28^. This rise may have been influenced by the drop in BCG vaccination rates across Brazil. Ireland, a high-income country with low tuberculosis rates, demonstrated a relationship between the decline in tuberculosis vaccination coverage and the increased burden of the disease^28^. In Brazil, Procianoy et al.^29^ identified a negative correlation between BCG vaccination coverage and the incidence of tuberculosis in children under one year of age. However, the researchers only conducted analyses between mean vaccination coverage rates at the national level, using a regression model more sensitive to time series analysis and identifying specific trend breakpoints. Conversely, our study conducted analyses at the regional level, allowing us to visualize the heterogeneity of these indicators in continental-sized Brazil.

Our study encountered some limitations. First, we relied on data from the SINAN, Brazil’s primary epidemiological surveillance system, which only records confirmed tuberculosis cases, excluding suspected cases. As previously stated, the paucibacillary nature of tuberculosis in the studied population, combined with the lack of laboratory tests and the difficulty in interpreting operator-dependent radiological tests, makes the diagnosis of tuberculosis in children particularly difficult^7^.This limitation may result in an underestimation of the true burden of tuberculosis among children and impact the cluster analysis. Additionally, case notifications are directly influenced by the training, motivation, and dedication of healthcare professionals. In some instances, demotivation and time constraints may lead to inadequate or incomplete reporting^22 , 30^. Our spatial analysis also revealed that many Brazilian states reported BCG vaccination rates above 120%, particularly from 2001 to 2006. This overestimation may be due to revaccination of children who did not develop a scar after their initial BCG vaccination^8^. Another limitation of this study is the effect of the epidemiological transition on pediatric tuberculosis. The epidemiological transition refers to the shift in health and disease patterns over time, driven by medical advances, improved living conditions, and demographic changes. This transition can influence the incidence and prevalence of infectious diseases such as tuberculosis. Ongoing socioeconomic and demographic factors, driven by this transition, can affect the distribution and detection of tuberculosis, complicating the accurate assessment of the disease’s spatial and temporal patterns. Thus, these factors must be considered when interpreting results and designing public health interventions for tuberculosis control^23^.

In Brazil, the strategy to combat tuberculosis includes early diagnosis, treatment, and BCG vaccination. Treatment is considered a key preventive measure as it mitigates the transmission of the disease^8^. Additionally, diagnosing tuberculosis in children is particularly difficult due to its paucibacillary nature in this population. As such, vaccination represents an essential measure to mitigate cases among children^8^.

In conclusion, our study identified a heterogeneous geographic distribution of childhood tuberculosis notification rates across Brazil. Amazonas had the highest incidence of tuberculosis in children, with certain municipalities forming clusters of cases. No significant movement of regional clusters was observed, suggesting that the states most affected by the disease have remained consistent over the past 20 years. Regarding BCG, we observed a progressive decrease in vaccination coverage over the last two decades, accompanied by an increase in tuberculosis cases in children under ten years of age since 2020. These findings emphasize the importance of tailored public policies in states such as Amazonas, Mato Grosso, and Rio de Janeiro, where a higher tuberculosis burden was observed, as these areas likely face unique challenges in controlling the disease.
